# *In vivo* Activity of Copper(II), Manganese(II), and Silver(I) 1,10-Phenanthroline Chelates Against *Candida haemulonii* Using the *Galleria mellonella* Model

**DOI:** 10.3389/fmicb.2020.00470

**Published:** 2020-03-24

**Authors:** Rafael M. Gandra, Pauraic McCarron, Livia Viganor, Mariana Farias Fernandes, Kevin Kavanagh, Malachy McCann, Marta H. Branquinha, André L. S. Santos, Orla Howe, Michael Devereux

**Affiliations:** ^1^Laboratório de Estudos Avançados de Microrganismos Emergentes e Resistentes, Departamento de Microbiologia Geral, Instituto de Microbiologia Paulo de Góes, Universidade Federal do Rio de Janeiro, Rio de Janeiro, Brazil; ^2^Instituto de Química, Programa de Pós-Graduação em Bioquímica, Universidade Federal do Rio de Janeiro, Rio de Janeiro, Brazil; ^3^Centre for Biomimetic and Therapeutic Research, Focas Research Institute, Technological University Dublin, Dublin, Ireland; ^4^Department of Biology, Maynooth University, National University of Ireland, Maynooth, Ireland; ^5^Department of Chemistry, Maynooth University, National University of Ireland, Maynooth, Ireland; ^6^School of Biological & Health Sciences, Technological University Dublin, Dublin, Ireland

**Keywords:** *Candida haemulonii*, metal-1, 10-phenanthroline chelates, *Galleria mellonella*, antifungal activity, immunomodulation

## Abstract

*Candida haemulonii* is an emerging opportunistic pathogen resistant to most antifungal drugs currently used in clinical arena. Metal complexes containing 1,10-phenanthroline (phen) chelating ligands have well-established anti-*Candida* activity against different medically relevant species. This study utilized larvae of *Galleria mellonella*, a widely used model of *in vivo* infection, to examine *C. haemulonii* infection characteristics in response to different copper(II), manganese(II), and silver(I) chelates containing phen, which had demonstrated potent anti-*C. haemulonii* activity in a previous study. The results showed that *C. haemulonii* virulence was influenced by inoculum size and incubation temperature, and the host *G. mellonella* immune response was triggered in an inoculum-dependent manner reflected by the number of circulating immune cells (hemocytes) and observance of larval melanization process. All test chelates were non-toxic to the host in concentrations up to 10 μg/larva. The complexes also affected the *G. mellonella* immune system, affecting the hemocyte number and the expression of genes encoding antifungal and immune-related peptides (e.g., *inducible metalloproteinase inhibitor protein*, *transferrin*, *galiomycin*, and *gallerimycin*). Except for [Ag_2_(3,6,9-tdda)(phen)_4_].EtOH (3,6,9-tddaH_2_ = 3,6,9-trioxoundecanedioic acid), all chelates were capable of affecting the fungal burden of infected larvae and the virulence of *C. haemulonii* in a dose-dependent manner. This work shows that copper(II), manganese(II), and silver(I) chelates containing phen with anti-*C. haemulonii* activity are capable of (i) inhibiting fungal proliferation during *in vivo* infection, (ii) priming an immune response in the *G. mellonella* host and (iii) affecting *C. haemulonii* virulence.

## Introduction

Invasive fungal infections have emerged as a significant global health problem with rising fungemia incidences associated with excessive morbidity and mortality (Castelli et al., 2014; Hamdy et al., 2016; Benedict et al., 2017). *Candida* is the most common fungal etiologic agent of blood infections (candidemia) related to vascular catheters, and is associated with mortality rates of approximately 35% and an impact of US$ 8 billion to the United States health-care system every year (World Health Organization, 2014). Invasive candidiasis affects more than a quarter of a million patients annually worldwide, and a candidemia incidence of 2–14 per 100,000 inhabitants in population-based studies have been described (Arendrup and Patterson, 2017). It is also problematic that recent data have demonstrated a shift on infections toward non-*albicans Candida* species that are more resistant to the available antifungal drugs (Arendrup and Patterson, 2017). This new and unsettling reality led the WHO to aim at establishing an operational framework to create a surveillance network for antifungal resistance as part of the previous established Global Antimicrobial Resistance Surveillance System (GLASS) (World Health Organization, 2019). Among such new resistant emerging opportunistic fungi is the *Candida haemulonii* complex (*C. haemulonii*, *Candida haemulonii* var. *vulnera*, and *Candida duobushaemulonii*) (Cendejas-Bueno et al., 2012; Kullberg and Arendrup, 2015). These species are highly resistant to azoles and polyenes, which are the most commonly prescribed antifungal drugs. *C. haemulonii* species are usually sensitive to echinocandins, a class of antifungal drugs acting on the inhibition of β-1,3-glucan synthase, which is a key enzyme for integrity of the fungal cell wall. However, reports of clinical isolates resistant to micafungin and of therapeutic failure with caspofungin have been cited (Khan et al., 2007; Kim et al., 2009; Muro et al., 2012). Such characteristics of the *C. haemulonii* species complex are a huge clinical threat and finding new compounds that are effective against such species is urgently required.

Transition metal chelates containing 1,10-phenantroline (phen) ligands have been shown to possess anti-*Candida* activity against species such as *Candida albicans, Candida glabrata, Candida Tropicalis*, and *C. krusei* (Geraghty et al., 2000; McCann et al., 2000, 2012; Coyle et al., 2003). These compounds affect mitochondrial function, reduce cytochrome *b* and *c* synthesis, induce respiratory uncoupling and increase cell wall permeability of *Candida* cells (McCann et al., 2004; Creaven et al., 2007). We have previously demonstrated that copper(II)-, manganese(II)-, and silver(I)-phen chelates are highly efficient against the *C. haemulonii* complex on both planktonic- and biofilm-growing cells (Gandra et al., 2017). These previous results stimulated the *in vivo* studies using *Galleria mellonella* larvae described herein, to further elucidate the hypothesis that such chelates could be potential alternative, metal-based drugs for treating *C. haemulonii* infections. The *in vivo G. mellonella* model has no ethical issues, is inexpensive and easy to manipulate and maintain. Most importantly, the innate immune system of *G. mellonella* possess several similarities with humans such as the similar lectin-mediated phagocytic mechanisms between hemocytes and neutrophils, respectively, identical cell surface receptors such as 3-glucan, identical signaling cascades of immune deficiency (IMD), c-Jun N-terminal kinases (JNK) and the Janus kinase/signal transducers and activators of transcription (JAK-STAT) pathways as well as nuclear factor-kappa B (NFkB) and IkB kinase transcription factors modulation by the toll and IMD pathways in the larvae and by the toll-like receptors and tumor necrosis factor alpha (TNF-α) in mammals (Browne et al., 2013; Wu et al., 2016). Moreover, a strong correlation involving microbial virulence and toxicity of novel antimicrobials between the *G. mellonella* and mammalian models has been established (Chamilos et al., 2007; Wang et al., 2013; Kavanagh and Sheehan, 2018). Therefore, the *G. mellonella* model has been widely used to assess the virulence of microbial pathogens and it was optimized to increase susceptibility to bacterial and fungal infections for testing the *in vivo* activity of antimicrobial agents (Kavanagh and Sheehan, 2018), which directly influenced our choice for this model in the current study. Corroborating these findings, recently, we showed that *G. mellonella* offered a simple and feasible model to study *C. haemulonii* complex virulence and drug efficacy, in which the first-line antifungals (fluconazole, amphotericin B, and caspofunfin) were tested with equivalent therapeutic doses and minimal inhibitory concentrations (MIC) profile *in vitro* was correlated with *in vivo* antifungal efficacy (Silva et al., 2018).

Herein, we further evaluate the chemotherapeutic potential of one copper(II)-, seven manganese(II)-, and three silver(I)-chelates containing the phen ligand toward the treatment of infections caused by a clinical isolate of *C. haemulonii* using the *in vivo* model, *G. mellonella.* We have investigated the virulence of the strain, how it affects the host immune system, how the chelates affect the ability of the fungal cells to infect a host and how the metal-chelates interfere with the larvae immune response.

## Materials and Methods

### *C. haemulonii* Strain

*Candida haemulonii* (LIP Ch4) isolated in the hospital service of Universidade Federal Fluminense (obtained from a fingernail) was used during this study. The antifungal susceptibility profile of the strain was previously described, being resistant to azoles and polyenes (Ramos et al., 2015; Silva et al., 2018). For all assays, fungal cells were incubated in Sabouraud dextrose culture medium for 48 h at 37°C under constant agitation (130 rpm). After culture, cells were centrifuged at 5,000 × *g* for 5 min, washed in sodium phosphate buffer (PBS; NaCl 150 mM, phosphate buffer 20 mM, pH 7.2) and cells estimated using a Neubauer chamber.

### *Galleria mellonella* Larvae

The larvae of the greater wax moth *G. mellonella* were purchased from Livefoods (Livefoods Direct, United Kingdom) and kept in wood shavings at 15°C in the dark. Larvae weighing between 0.2 and 0.3 g with no color alterations were selected for all experiments and used in groups of 10 per experimental parameter for mortality and 3 for hemocyte extractions (Bergin et al., 2003).

### Metal Chelates

The metal chelates used during this study were prepared following previously published methods (Casey et al., 1994; McCann et al., 1997, 2004; Devereux et al., 2000; Leon, 2000; Gandra et al., 2017) and are summarized in Table 1.

**TABLE 1:** Copper(II) (1), manganese(II) (2-8), and silver(I) (9-11) chelates.

**Chelate**	**Formula**	**Synthesis references**
1	{[Cu(3,6,9-tdda)(phen)**_2_**].3H**_2_**O.EtOH}***_n_***	Gandra et al., 2017
2	[Mn(ph)(phen)(H_2_O)_2_]	Devereux et al., 2000
3	[Mn(ph)(phen)_2_(H_2_O)].4H_2_O	Devereux et al., 2000
4	[Mn_2_(isoph)_2_(phen)_3_].4H_2_O	Devereux et al., 2000
5	{[Mn(phen)_2_(H_2_O)_2_]}_2_(isoph)_2_(phen).12H_2_O	Devereux et al., 2000
6	[Mn(tereph)(phen)_2_].5H_2_O	Leon, 2000
7	[Mn_2_(oda)(phen)_4_(H_2_O)_2_][Mn_2_(oda)(phen)_4_ (oda)_2_].4H_2_O	Casey et al., 1994
8	{[Mn(3,6,9-tdda)(phen)_2_].3H_2_O.EtOH}_n_	McCann et al., 1997
9	[Ag(phendione)_2_]ClO_4_	McCann et al., 2004
10	[Ag**_2_**(3,6,9-tdda)(phen)**_4_**].EtOH	Gandra et al., 2017
11	[Ag(phen)_2_]ClO_4_	McCann et al., 2004

**3,6,9-tddaH_2_, 3,6,9-trioxaundecanedioic acid; phH_2_, phthalic acid; isophH_2_, isophthalic acid; terephH_2_, terephthalic acid; odaH_2_, octanedioic acid; phen, 1,10-phenanthroline; phendione, 1,10-phenanthroline-5,6-dione*.*

### Effects of the Metal Chelates on *C. haemulonii*: Fungicidal and Fungistatic Activity

*Candida haemulonii* was inoculated in a 96-well plate (10^6^ cells/well) containing 200 μL of culture medium and the chelates with concentrations ranging from 2 to 0.125 mg/L. Plates were incubated at 37°C for 24 h and the contents of each well collected in Eppendorf tubes and centrifuged at 12,000 *g* for 2 min. The supernatant was discarded, the cell pellet re-suspended in PBS and cellular density was established by direct count in a Neubauer chamber. The chelate concentration capable of reducing cellular density by 50% was established by plotting the number of viable cells vs. the drug concentration log using the Origin Pro 7.5 software. The microdilution protocol described in the document, M27-A3 published by the Clinical Laboratory Standards Institute, was used for the fungicidal vs. fungistatic activity analysis (CLSI, 2008). *C. haemulonii* (10^6^ cells/well) was inoculated into a 96-well plate containing the chelates in concentrations ranging from 0.0625 to 32 mg/L and plates were incubated for 48 h at 37°C. After that, an aliquot of 50 μl from each well with no visible growth was inoculated into agar Sabouraud plates, which were incubated for 48 h at 37°C. The plates where visible colonies were present were considered as a fungistatic chelate concentration, while complete absence of colonies were designated a fungicidal concentration.

### Fungal Infection of *Galleria mellonella* Larvae

The infection profile was assessed using a previously described methodology (Desbois and Coote, 2012; Kavanagh and Sheehan, 2018). *C. haemulonii* cells were suspended in PBS in the following concentrations: 5 × 10^4^, 5 × 10^5^, 5 × 10^6^, 5 × 10^7^, and 5 × 10^8^ cells/mL. Using a Myjector syringe (23 gauge; BD PrecisionGlide), 10 larvae were inoculated through the last left pro-leg with 20 μl of the cell suspensions, with final concentrations of 10^3^, 10^4^, 10^5^, 10^6^, and 10^7^ fungal cells/larvae, which were injected directly into the hemocoel. Untouched larvae and larvae inoculated with 20 μL of sterile PBS were used as controls. The larvae were placed in sterile petri dishes containing 9 mM filter paper and wood shavings and incubated at 30 or 37°C for up to 9 days, and the mortality rate was evaluated every 24 h post-infection. For virulence comparison purposes, a *C. albicans* strain (ATCC 10231) was used under the same conditions. Death was determined by melanization and lack of movement after stimulation with a needle (Barnoy et al., 2017).

### Fungal Burden

Larvae were inoculated with different *C. haemulonii* concentrations ranging from 10^3^ to 10^7^ cells per larvae. The infected models were incubated at 30°C, and after 6, 24, and 48 h of infection 3 larvae were randomly selected and washed in ethanol 70%. After drying, the larvae were cut into small pieces using a sterile scalpel and added to 15 mL falcon tubes containing 1 mm glass beads (1 g) and 1 mL of PBS + ampicillin (1 mg/L). The tubes were vortexed for 10 sec, and 100 μL of each sample was collected and serially diluted. The dilutions were plated (50 μL) onto Sabouraud plates containing chloramphenicol (100 mg/L). The plates were incubated during 48 h at 30°C and the number of colony forming units (CFU) were counted.

### *G. mellonella* Immune Response to Fungal Infection

Larvae were inoculated with different *C. haemulonii* concentrations ranging from 10^3^ to 10^7^ cells per larvae. The infected models were incubated at 30°C, and after 6, 24, and 48 h of infection 3 larvae were randomly selected and the haemolymph extracted (2–3 drops) by piercing the back of the anterior end with a sterile needle. To prevent clotting and melanization, the haemolymph was collected into a pre-chilled Eppendorf and 30 μL were transferred into a 1 in 10 solution of cold PBS containing 0.37% (v/v) 2-mercaptoethanol (Sigma-Aldrich) (Kelly and Kavanagh, 2011). After gentle homogenization, 10 μL of the solution were transferred to a Neubauer chamber and the hemocytes number counted under a microscope. Three different counts were performed for each sample and the cell number multiplied by the dilution factor of the haemolymph (10) and the hemocytometer (50.000) in order to determine cellular density.

### *G. mellonella* Larvae Response to Metal-Chelates

#### Metal-Chelate Toxicity and Effect on *G. mellonella* Immune Response

Each chelate solution was diluted in dimethyl sulfoxide (DMSO) or sterile water in concentrations of 1,500, 750, 500, 200, and 100 mg/L. Using a Myjector syringe (23 gauge; BD PrecisionGlide), groups of 10 larvae were inoculated with 20 μL of the diluted working solutions, resulting in final test concentrations of 30, 15, 10, 4, and 2 μg/larva. Untouched larvae, larvae inoculated with DMSO equivalent to the highest concentration present in the dilutions (2%) and larvae inoculated with 20 μL of sterile PBS were used as controls. The injected samples were placed in sterile petri dishes containing 9 mM filter paper and wood shaves and incubated at 30°C for 72 h and mortality rate was evaluated every 24 h. For the immunomodulation assays, groups of 10 larvae were inoculated with 15 and 30 μg/larvae of the chelates and incubated for 24 h as previously described. After this period, 3 random larvae were chosen, the haemolymph extracted and hemocytes density established as described above (Kelly and Kavanagh, 2011).

#### Metal-Chelate Influence Over Expression of Relevant Antimicrobial Peptides

##### Target genes and primer sequences

Primer sets (Forward and Reverse sequences) for target and reference genes were obtained from Sigma Genosys. Table 2 indicates the primer sequences for the gene *S7e*, a housekeeping gene that encodes the ribosomal protein S7e and the 4 target genes associated with the *G. mellonella* immune response, namely *transferrin*, *IMPI*, *galiomicin*, and *Gallerimycin*. In the analysis of each target gene, it was normalized to the reference gene.

**TABLE 2:** Forward (F) and reverse (R) sequences of genes analyzed.

**Primer name**	**Oligonucleotides (5′–3′)**	**Fragment size [base pair (bp)]**
S7e F S7e R	ATGTGCCAATGCCCAGTTG GTGGCTAGGCTTGGGAAGAAT	131
Transferrin F Transferrin R	CCCGAAGATGAACGATCAC CGAAAGGCCTAGAACGTTTG	535
IMPI F IMPI R	ATTTGTAACGGTGGACACGA CGCAAATTGGTATGCATGG	409
Galiomicin F Galiomicin R	CCTCTGATTGCAATGCTGAGTG GCTGCCAAGTTAGTCAACAGG	359
Gallerimycin F Gallerimycin R	GAAGATCGCTTTCATAGTCGC TACTCCTGCAGTTAGCAATGC	175

*The housekeeping gene S7e was used as reference.*

##### RNA extraction of *G. mellonella* exposed to metal chelates

Groups of 10 larvae were exposed to the highest concentration in which none of the chelates induced mortality (10 μg/larvae) by inoculation directly into the larvae haemolymph as previously described. All workspace and equipment were previously treated with RNase Away (Molecular Bioproduct). After 24 h, 3 larvae were randomly chosen and immersed in liquid nitrogen in a sterile mortar and ground-up with a sterile pestle into a fine powder. The mortar was allowed to warm for a few minutes and 1 mL of Tri-reagent (Sigma-Aldrich) was added and the resulting mixture was gently transferred to an Eppendorf. The sample was allowed to stand for 2 min before being centrifuged at 2,000 *g* for 2 min and the supernatant was transferred to a new Eppendorf while the pellet was discarded. The samples were either stored at −80°C or immediately used. To extract the RNA, samples were thawed and gently inverted and chloroform (Sigma-Aldrich) was added to the tubes. After 5 min the samples were centrifuged for 15 min at 12,000 *g* at 4°C to separate it into 3 distinct phases. The upper phase (clear) containing the RNA was carefully transferred to a separate Eppendorf. Propan-2-ol (Sigma) (0.5 mL) was added and the tubes were gently inverted and incubated at room temperature for 10 min and then centrifuged at 4°C for 10 min at 12,000 *g*. The supernatant was removed and the RNA pellet was washed once with 1 mL of 75% ethanol (Sigma) and then re-suspended in 30 μL of 0.1% dietyl pyrocarbonate (DEPC) treated water. The purity and concentration of the RNA samples was measured using the MaestroNano^TM^ spectrophotometer (MaestroGen, United States). After an initial blank with 0.1% DEPC treated water, the absorbance ratios (A260/A280 and A260/A230) of the samples were recorded. The samples were then stored at −80°C.

##### Real-Time, One-step quantitative RT-PCR

Real-Time, One-step RT-PCR was performed in triplicate using a QuantiFast SYBR Green RT-PCR Kit (Qiagen, United Kingdom) following the manufactures guidelines. The reactions were carried out in a 25 μL total volume containing 100 ng of total RNA and 1 μM final concentration for each forward and reverse primer in a 96-well plate. Each plate contained negative controls (without RNA or without RT mix). The plates were sealed and centrifuged at 1,200 *g* for 2 min before being placed in a 7,500 Fast Real-Time PCR System (Applied Biosystems, Foster City, CA, United States). The Real-Time cycler conditions included 10 min RT reaction at 50°C and 5 min PCR initial activation step for 5 min at 95°C, followed by 40 cycles of denaturation during 10 s at 95°C and 30 s of combined annealing/extension at 60°C. After that, melting-curve analysis were performed to confirm RT-PCR specificities. The relative expression was calculated using the 2^–ΔΔ^*^C^*^t^ method, and the *C*t values of all immune-related genes analyzed were normalized against the expression of the *S7e* reference gene.

### Metal-Chelates Treatment Effect Over Fungal Burden on *G. mellonella* Infected With *C. haemulonii* and Pre-treatment Effect Over Mortality

Groups of 10 larvae of *G. mellonella* were inoculated with 5 × 10^5^ cells of *C. haemulonii* per larvae as previously described. After 1 h post-inoculation, the larva were treated with the chelates in different concentrations (5, 2.5, 1.25, and 0.625 μg/larva). Untouched larvae, larvae inoculated with PBS and larvae infected with *C. haemulonii* but not treated were used as a control. The samples were then incubated at 30°C, and the resulting fungal burden analyzed after 24 and 48 h of infection. For the fungal burden analysis, 3 larvae were randomly selected and washed in ethanol (70% v/v). After they had dried up, the larvae were cut into small pieces using a sterile scalpel and added to 15 mL falcon tubes containing 1 mm glass beads (1 g) and 1 mL of PBS + ampicillin (1 mg/L). The tubes were vortexed for 10 s and samples were diluted up to 100.000×. Diluted samples were plated (50 μL) into Sabouraud plates containing chloramphenicol (100 mg/L). The plates were incubated for 48 h at 30°C and the number of CFU were counted (Mesa-Arango et al., 2013). For the pre-treatment impact over virulence assays, *C. haemulonii* cells (5 × 10^6^) were incubated in Sabouraud broth containing the chelates in two different sub-inhibitory concentrations (1/2 and 1/4 of the IC_50_ value) for 24 h at 37°C. After the exposition period, the tubes were centrifuged at 14,000 *g* for 2 min and cells were washed twice in PBS, being resuspended in 20 μL of PBS and inoculated into the larvae as previously described, with mortality being assessed after 24 and 48 h post-infection.

### Statistical Analysis

All experiments were performed in triplicate, in three independent experimental sets. Data were analyzed statistically by means of Student’s *t*-test using the GraphPad Prism version 5.00 for Windows, GraphPad Software (La Jolla, CA, United States). In all analyses, *P*-values of 0.05 or less were considered statistically significant.

### Ethics Statement

An ethics statement is not necessary. The clinical isolate was not obtained directly from a patient. The sample was received anonymized, without the name or any personal information from the source. No vertebrates or higher vertebrate animals were used during the study.

## Results

### Metal-Chelate IC_50_ Values and Fungistatic/Fungicidal Activity

The IC_50_ values of *C. haemulonii* exposed to the chelates (Table 3) ranged between 0.36 and 1.07 mg/L. Silver(I) chelates had better activity in general, with values ranging between 0.36 and 0.77 mg/L, while manganese(II) chelates had IC_50_ values ranging between 0.55 and 1.07 mg/L. Fungicidal activity occurred in concentrations ranging from ≥4 to 32 mg/L for manganese(II), ≥1 to ≥8 for silver(I) chelates, and of ≥8 for the copper(II) chelate. The best fungicidal activity was observed for the silver(I) chelates 9 and 11. The manganese(II) chelate, 6, on the other hand, only demonstrated fungicidal activity at 32 mg/L.

**TABLE 3:** IC_50_ and fungicidal concentration values for *C. haemulonii* exposed to metal chelates.

**Metal chelate**	**IC_50_**	**Fungicidal**^a^
	**(mg/L)**	**(mg/L)**
{[Cu(3,6,9-tdda)(phen)_2_]3H_2_O.EtOH}_n_ (1)	0.52 ± 0.08	8
[Mn(ph)(phen)(H_2_O)_2_] (2)	0.96 ± 0.23	8
[Mn(ph)(phen)_2_(H_2_O)]4H_2_O (3)	0.78 ± 0.18	4
[Mn_2_(isoph)_2_(phen)_3_]4H_2_O (4)	0.69 ± 0.01	16
{[Mn(phen)_2_(H_2_O)_2_]}_2_(isoph)_2_(phen).12H_2_O (5)	1.07 ± 0.24	8
[Mn(tereph)(phen)_2_]5H_2_O (6)	0.59 ± 0.10	32
[Mn_2_(oda)(phen)_4_(H_2_O)_2_][Mn_2_(oda) (phen)_4_(oda)_2_]4H_2_O (7)	0.55 ± 0.03	8
{[Mn(3,6,9-tdda)(phen)_2_]3H_2_O.EtOH}_n_ (8)	0.65 ± 0.10	4
[Ag(phendione)_2_]ClO_4_ (9)	0.77 ± 0.02	2
[Ag_2_(3,6,9-tdda)(phen)_4_].EtOH (10)	0.39 ± 0.07	8
[Ag(phen)_2_]ClO_4_ (11)	0.36 ± 0.08	1

**^a^Values reflect concentrations at which no cell growth was detected in the wells after visual inspection and after the sample being plated into sabouraud agar and incubated for 48 h*.*

### *C. haemulonii* Virulence to *G. mellonella*

**FIGURE 1:**
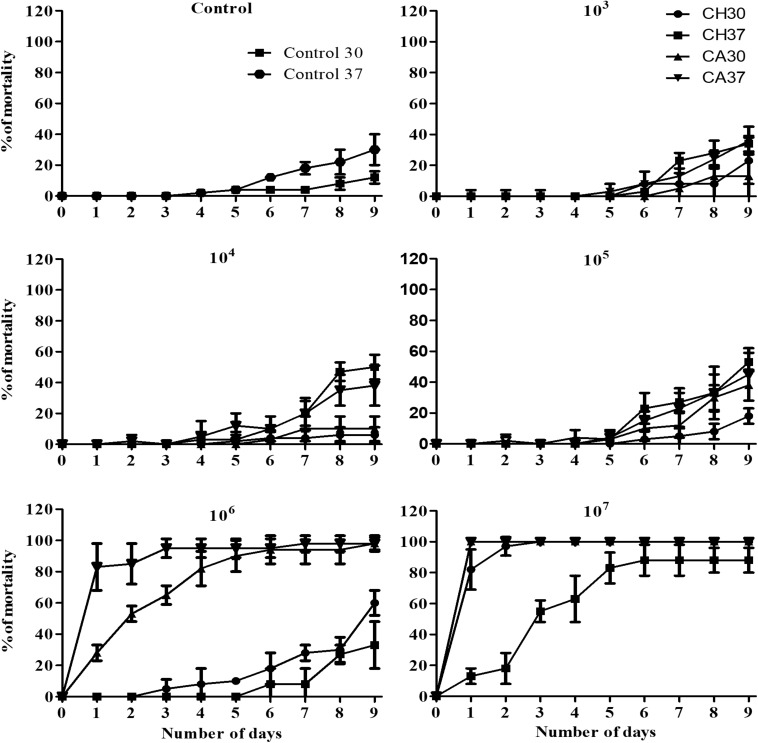
Evaluation of the virulence of *Candida haemulonii* and temperature effect over pathogenesis on *Galleria mellonella*. *Candida Haemulonii*, and *Candida albicans* cells incubated at 37°C for 48 h were harvested and inoculated into groups of 10 larvae of *G. mellonella* in different concentrations (10^3^–10^7^ cells/larvae). Infected larvae were incubated at 30 or 37°C for up to 9 days and mortality assessed every 24 h. CH 30, *C. haemulonii* 30°C; CA 30, *C. albicans* 30°C; CH 37, *C. haemulonii* 37°C; CA 37, *C. albicans* 37°C. Larvae inoculated with PBS were used as control. Mortality was determined by lack of movement after stimulation with a needle and also by melanization.

**FIGURE 2:**
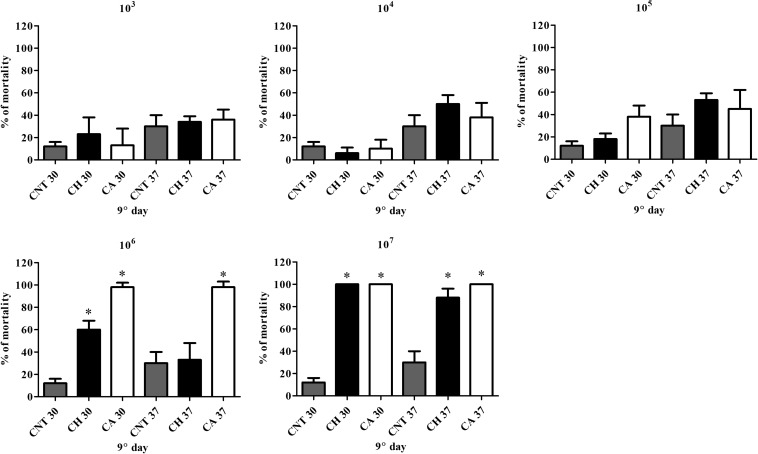
Comparison of mortality induced by *C. albicans* and *C. haemulonii* in *G. mellonella*. Larvae inoculated with different *C. haemulonii* cell concentrations had their mortality assessed after 9 days of incubation at 30 and 37°C. Larvae inoculated with *C. albicans* under the same conditions were used for comparison. The asterisks denote significant differences compared to the control (^∗^*P* < 0.05). The bars represent the mean ± standard of three independent experiments. CNT, control (gray bars); CH, *C. haemulonii* (black bars); CA, *C. albicans* (white bars).

Larvae infected with *C. haemulonii* and with *C. albicans* under the same conditions and incubated at 30 or 37°C were used in this assay (Figures 1, 2). Larvae infected with 10^3^–10^5^ fungal cells/larvae had a small mortality rate after 5 days of incubation, but with no statistical relevance when compared to the control inoculated only with PBS. The inoculum of 10^6^ cells/larvae of *C. albicans* induced mortality rates of 28% after 24 h and 53, 65, 82, and 90% on the following days at 30°C. In contrast, the systems incubated at 37°C demonstrated mortality rates of 83, 85, and 95% after 24, 48, and 72 h post-infection, respectively. At all analysis points, mortality was statistically significant when compared with the PBS-inoculated control (*p* < 0.05, student *t-*test). The 10^7^ cells/larva inoculum caused 100% mortality after 24 h at both temperatures. Regarding *C. haemulonii*, mortality in larvae infected with 10^6^ cells and incubated at 30°C was observed after 3 days, which gradually increased and was statistically significant after 8 days (30% mortality) when compared with the PBS-inoculated control. Larvae incubated at 37°C only demonstrated mortality after 6 days, but with no statistical relevance when compared with the PBS-inoculated control. When the inoculum was increased to 10^7^ cells/larvae, animals incubated at 30°C had mortality rates of 82% after 24 h, increasing to 97 and 100% after 48 h and 72 h, respectively. Systems incubated at 37°C had mortality rates of 13% after 24 h, progressively increasing to 18, 55, 63, 83, and 88% each day. After 9 days, fungal inoculums of 10^3^ to 10^5^ cells/larvae of both species were not capable of significantly affecting the larvae mortality. *C. albicans* induced mortality rates of 98% (10^6^ inoculum) and 100% (10^7^) at both 30 and 37°C. The *C. haemulonii* 10^6^ inoculum only induced statistically relevant mortality at 30°C (60%), while the inoculum of 10^7^ cells/larvae resulted in mortality rates of 100% at 30°C and 88% at 37°C. In the control systems (PBS-inoculated), mortality rates of 30% were observed at 37°C and of 12% at 30°C.

### Melanization Process in Infected Larvae

**FIGURE 3:**
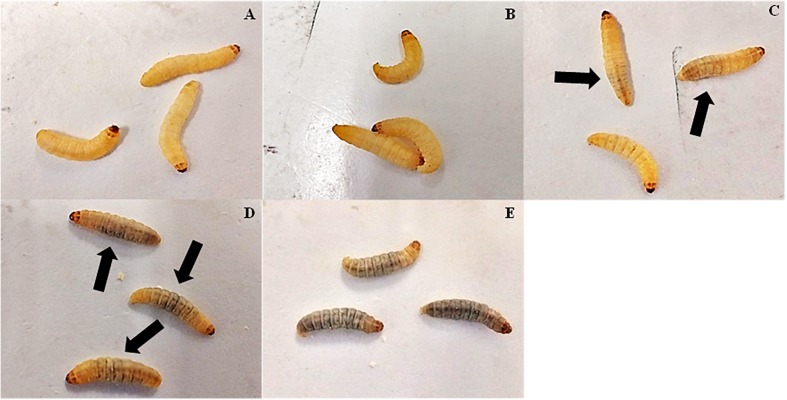
Melanization process in *G. mellonella* infected with *C. haemulonii.* Larvae infected with different *C. haemulonii* inoculums (**(A)** 10^3^, **(B)** 10^4^, **(C)** 10^5^, **(D)** 10^6^, and **(E)** 10^3^ cells/larvae) were incubated for 1 h at 30°C and photographed. The arrows indicate melanized areas observable in the images.

No cuticle color alterations were observed in *C. haemulonii* larvae inoculate with 10^3^ and 10^4^ cells/larvae (Figures 3A,B). The 10^5^ inoculum resulted in small-darkened areas around the needle insertion area (Figure 3C). Larvae infected with 10^6^ cells had more pronounced alterations, with larger darkened areas observable (Figure 3D). An intense and disseminated melanization process was observed in larvae infected with the 10^7^ inoculum (Figure 3E).

### Fungal Burden Determination in *G. mellonella*

**FIGURE 4:**
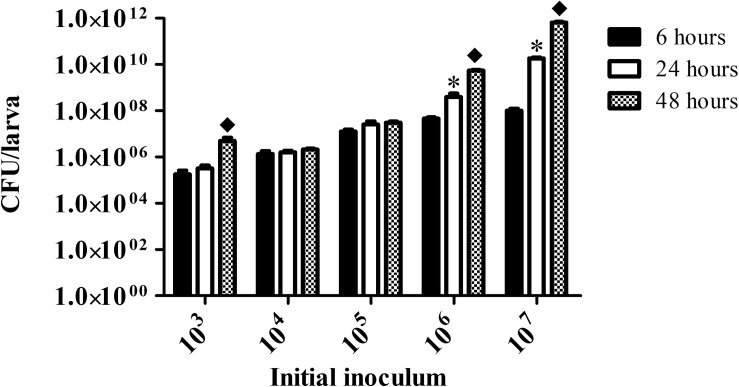
Fungal burden in infected *G. mellonella*. Larvae infected with different *C. haemulonii* inoculum sizes were incubated at 30°C for up to 48 h. At 6, 24, and 48 h incubation points, 3 random larvae were collected, sliced with a scalpel and macerated. The extract was then diluted and plated into agar sabouraud plates containing chloramphenicol. Plates were incubated for 48 h and the resulting number of colonies were counted. The asterisks denote significant differences between the 6 and 24 h point (^∗^*P* < 0.05). The diamond symbol denotes significant differences between the 24 and 48 h point (◆*P* < 0.05). The bars represent the mean ± standard of three independent experiments.

Fungal burden in larvae infected with 10^3^ *C. haemulonii* cells remained stable between the 6 and 24 h analysis points, but a significant increase was observed from 24 to 48 h of incubation (*P* = 0.04) (Figure 4). It remained stable in all analysis points in animals infected with the initial inoculums of 10^4^ and 10^5^ cells/larvae, indicating that the infection was kept under control by the larvae immune system. Increased infection progression and fungal burden was detected in an inoculum-dependent manner. Infecting animals with 10^6^ cells resulted in fungal burden progression from 6 h to 24 h (*P* = 0.01) and from 24 to 48 h (*P* = 0.003). Such increases were even more pronounced with the 10^7^ inoculum, with significant progression from 6 to 24 h (*P* = 0.002) and from 24 to 48 h (*P* = 0.001).

### Immune Modulation Induced by *C. haemulonii* Infection

**FIGURE 5:**
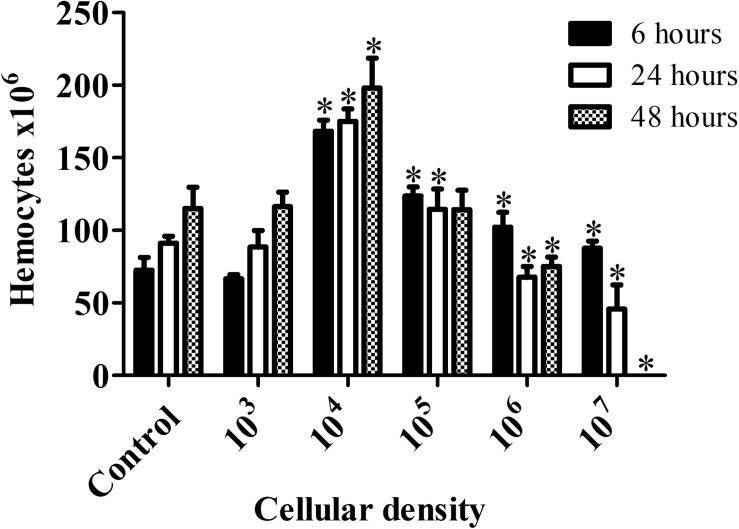
*Galleria mellonella* immumodulation induced by *C. haemulonii* infection. Larvae were inoculated with different *C. haemulonii* inoculum sizes and incubated at 30°C. Hemocytes were extracted and counted using a Neubauer chamber after 6, 24, and 48 h of incubation. The asterisks denote significant differences compared to the control (^∗^*P* < 0.05). The bars represent the mean ± standard of three independent experiments.

Hemocyte count for larvae infected with *C. haemulonii* indicated the presence of an inoculum-dependent immune response (Figure 5). No alterations in animals exposed to 10^3^ cells/larva were detected during the whole analysis. The highest immune response was observed with the 10^4^ inoculum, which significantly increased hemocyte density by 132.13% (6 h), 91.88% (24 h), and 72.26% (48 h) when compared with the PBS-inoculated control. The 10^5^ inoculum resulted in increased levels of hemocytes during the first 48 h, while the 10^6^ and 10^7^ inoculums increased density after 6 h, followed by a significant drop as time progressed. Due to high mortality rates, it was not possible to estimate hemocytes density on larvae infected with 10^7^ cells after 48 h.

### Metal-Chelate Toxicity Evaluation Using *G. mellonella* Larvae

After 72 h of exposure to the chelates, a dose-dependent toxicity to *G. mellonella* was detected. All animals tolerated doses of 2, 4, and 10 μg/larvae (Table 4), with no mortality observed. Similarly, chelates 4, 6, 7, 8, 10, and 11, at the concentration of 15 μg/larvae, induced no mortality, while the remaining chelates resulted in 10% (9), 13.33% (5), 35% (3), 40% (2), and 60% (1) mortality. Increasing the chelate concentration to 30 μg/larvae resulted in mortality rates ranging between 80 and 100%, except for chelates 7 (30%), 8 (13.33%), 10 (33.33%), and 11 (20%).

**TABLE 4:** Chelate toxicity toward *G. mellonella*.

**Chelate**	**2 μg (μM)**	**4 μg (μM)**	**10 μg (μM)**	**15 μg (μM)**	**30 μg (μM)**
1	0 ± 0(2.68)	0 ± 0(5.36)	0 ± 0(13.41)	60 ± 14.14(20.15)	100 ± 0(40.30)
2	0 ± 0(4.6)	0 ± 0(9.18)	0 ± 0(22.97)	40 ± 0(34.46)	90 ± 0(68.92)
3	0 ± 0(2.97)	0 ± 0(5.94)	0 ± 0(14.86)	35 ± 7.07(22.30)	96.67 ± 5.77(44.6)
4	0 ± 0(1.90)	0 ± 0(3.80)	0 ± 0(9.51)	0 ± 0(14.27)	80 ± 0(28.54)
5	0 ± 0(1.22)	0 ± 0(2.44)	0 ± 0(6.14)	13.33 ± 5.77(9.21)	90 ± 0(18.42)
6	0 ± 0(2.98)	0 ± 0(5.96	0 ± 0(14.93)	0 ± 0(22.40)	85 ± 7.07(44.8)
7	0 ± 0(0.81)	0 ± 0(1.62)	0 ± 0(4.06)	0 ± 0(6.10)	30 ± 0(12.2)
8	0 ± 0(2.71)	0 ± 0(5.42)	0 ± 0(13.59)	0 ± 0(20.39)	13.33 ± 5.77(40.78)
9	0 ± 0(1.66)	0 ± 0(3.32)	0 ± 0(8.31)	10 ± 10(12.47)	95 ± 7.07(24.94)
10	0 ± 0(3.52)	0 ± 0(7.04)	0 ± 0(17.61)	0 ± 0(26.42)	33.33 ± 5.77(52.84)
11	0 ± 0(4.65)	0 ± 0(9.30)	0 ± 0(23.25)	0 ± 0(34.88)	20 ± 14.14(68.75)

**Values and percentages expressed refers to the% of total mortality in a system containing 10 larvae after 72 h of exposition to the chelates. The values in parenthesis represents the chelate concentration in micromolar/larvae*.*

### Immunomodulation Induced by the Chelates

**FIGURE 6:**
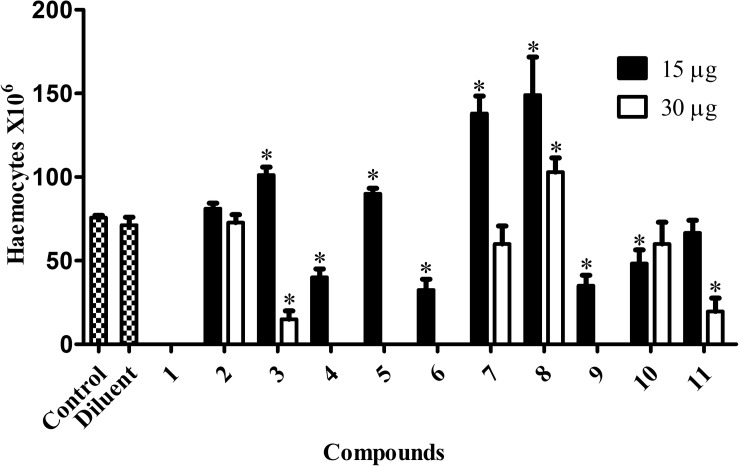
Immunomodulation induced by the chelates in *G. mellonella. G. mellonella* larvae were exposed to the metal chelates in different concentrations (15 and 30 μg/larva) during 24 h at 30°C. After incubation, 3 larvae were randomly chosen, their hemocytes extracted and the number estimated. The bars represent the mean ± standard deviation of three independent experiments. Asterisks indicate significant differences in relation to the control system (*P* < 0.05).

Control larvae (not exposed to any chelate) had a median hemocyte density of 75.8 × 10^6^ cells/mL (Figure 6). At an administered chelate concentration of 15 μg/larva, animals exposed to chelates 3 (101.2 × 10^6^/mL), 5 (90 × 10^6^/mL), 7 (138 × 10^6^/mL), and 8 (149 × 10^6^/mL) had significantly elevated hemocyte density. No significant differences were detected with chelates 2 and 11. Doubling the concentration to 30 μg/larva resulted in a reduction in hemocytes for chelates 3 (15 × 10^6^/mL) and 11 (19.6 × 10^6^/mL) and an increase with chelate 8 (103 × 10^6^/mL). Chelates 2, 7, and 10 did not induce significant alterations, whilst chelates 4, 5, 6, and 9, at 30 μg/larva, negated all hemocytes. There was a complete absence of hemocytes when chelate 1 was used at both concentrations. The toxicity profiles of the chelates could be partially responsible for the hemocyte density alterations. Chelates used at 30 μg/larva that induced 0% mortality (7 and 8) induced higher hemocyte density or did not affect it (11), while the more toxic chelates (9, 6, 10, and 4) led to a significant reduction in hemocyte production.

### Metal-Chelate Effect on the Expression of Antimicrobial Peptides in *G. mellonella*

**FIGURE 7:**
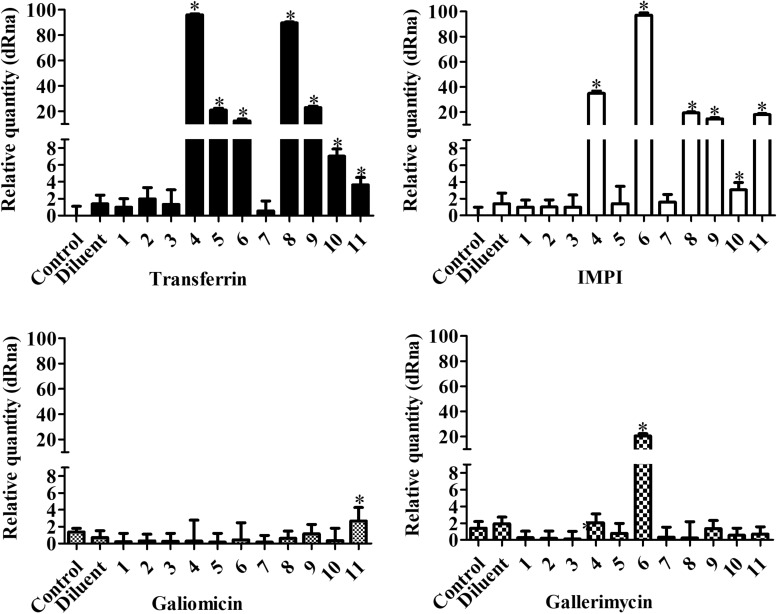
Effect of chelates on antimicrobial peptide expression in *G. mellonella*. Larvae were inoculated with 10 μg/larva of the chelates and incubated for 24 h at 30°C. After that, total RNA was extracted and RT-PCR performed. The genes *transferrin*, *IMPI*, *galiomycin*, and *gallerimycin* were analyzed and PCR products were normalized against the housekeeping gene, *S7e*. The bars represent the mean ± standard deviation of three independent experiments. Asterisks indicate significant differences in relation to the control system (*P* < 0.05).

The expression of the immune related peptide *genes transferrin*, *IMPI*, *galiomicin*, and *gallerimycin* in *G. mellonella* exposed to the metal-chelates was normalized against the *S7e* reference gene (Figure 7). *Transferrin* and *IMPI* expression was significantly affected by the manganese(II) chelates 4, 5, 6, and 8 and silver(I) chelates 9, 10, and 11 (*P* < 0.05). *Galiomicin* expression was increased by the silver(I) chelate 11, while *gallerimycin* expression was increased by the manganese(II) chelates 4 and 6 (*P* < 0.05). It is noteworthy that, besides affecting *IMPI* and *transferrin* expression, the manganese(II) chelates 5 and 8 are capable of increasing hemocyte density in *G. mellonella*, as previously described.

### Treatment Effect Over Fungal Burden in Infected Larvae of *G. mellonella*

**FIGURE 8:**
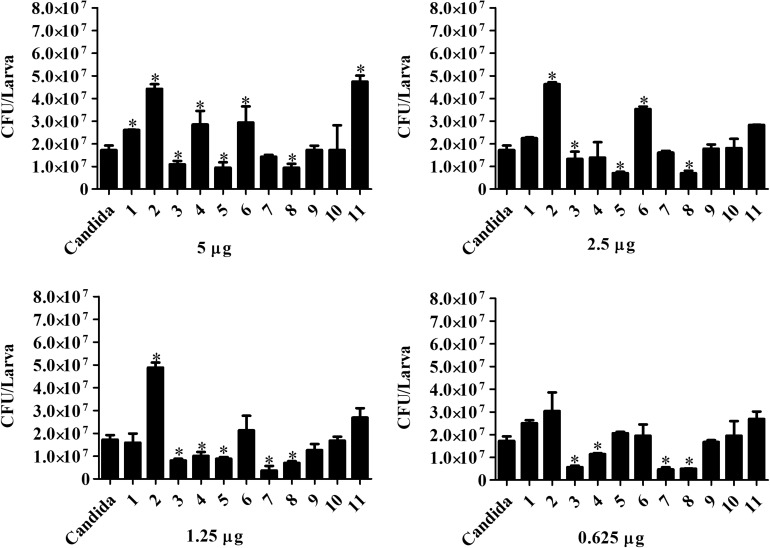
Effect of chelate treatment (24 h) on fungal burden in infected larvae of *G. mellonella*. Larvae infected with 5 × 10^5^ cells/larva of *C. haemulonii* were treated with the chelates 1 h post-infection. The larvae extract was plated into agar sabouraud containing chloramphenicol and the resulting number of colonies counted. Asterisks indicate significant differences in relation to the control system (*P* < 0.05).

**FIGURE 9:**
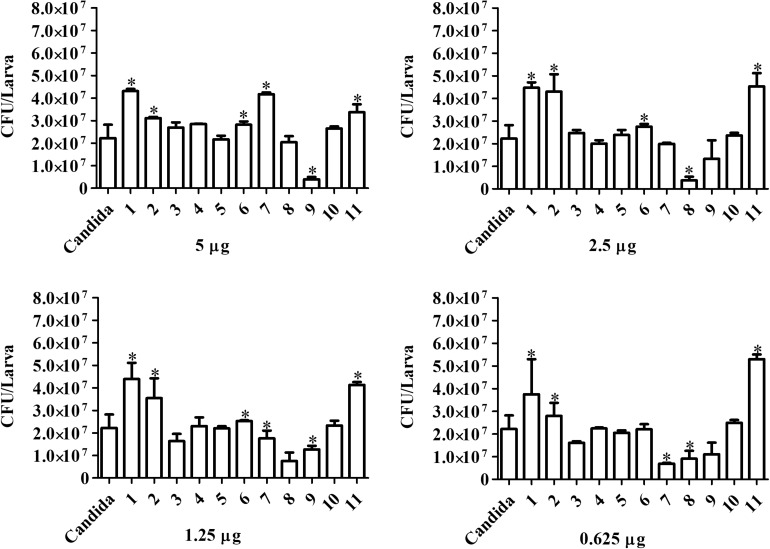
Forty-eight hours treatment effect over fungal burden in infected larvae of *G. mellonella*. Larvae infected with 5 × 10^5^ cells/larvae of *C. haemulonii* and treated with different chelates concentration 1 h post-infection were macerated, and the extract diluted and plated into agar sabouraud plates containing chloramphenicol. Asterisks indicate significant differences in relation to the control system (*P* < 0.05).

*Candida haemulonii-*infected *G. mellonella* were treated with the chelates in different concentrations 1 h post-infection. Analysis, after 24 h of treatment (Figure 8), indicates that chelates 3 and 8 significantly reduced the number of yeast cells present in the infected larvae at all of the chelate concentrations used. Chelate 5 reduced fungal burden at all concentrations except 0.625 μg/larva, while chelates 4 and 7 reduced fungal burden at the two lowest test concentrations. An increase in the fungal burden was observed with chelates 4 and 11 at the highest concentration, with chelate 6 at 5 and 2.5 μg/larva and with chelate 2 across all administered concentrations. No significant differences were observed with the other chelates. Analysis at the 48 h post-treatment time revealed that only chelates 7 and 8 retained the ability to inhibit the fungal proliferation observed at 24 h post-infection (Figure 9). All of the other chelates increased fungal burden or did not affect the number of fungal cells retrieved, except for compound 9.

### Pre-treatment Effect Over *C. haemulonii* Virulence

*Candida haemulonii* cells were exposed to sub-inhibitory concentrations of the chelates (1/2 × IC_50_ and 1/4 × IC_50_) for 24 h before inoculation of *G. mellonella* with these fungal cells. The analysis, 24 h post-infection, revealed that non-treated fungal cells caused 100% larvae mortality, while some of the chelates were capable of affecting the fungal virulence (Table 5). For *C. haemulonii* pre-treated with 1/2 × IC_50_, the manganese(II) chelates 2, 3, 5, and 6 and silver(I) chelate 11 were the most effective, reducing mortality rates by 55–100%. Lowering the concentration of pre-administered chelates to 1/4 × IC_50_ resulted in no alterations in virulence with chelates 1 and 7 while the other chelates reduced mortality by 10–55%. After 48 h of infection by the pre-treated *C. haemulonii* cells, larvae mortality rates were high (75–100%) for all chelates except 3 and 5 (90% reduction in mortality with 1/2 × IC_50_).

**TABLE 5:** Impact on virulence of *Candida haemulonii* toward *G. mellonella* following pre-exposure of the fungal cells to sub-inhibitory concentrations of the chelates.

**Chelate**	**Time of infection (hours)**			
	**24**		**48**	
Control	0 ± 0		0 ± 0	
*Candida haemulonii*	85 ± 7.07		100 ± 0	

**Concentration**	**1/2 IC_50_**	**1/4 IC_50_**	**1/2 IC_50_**	**1/4 IC_50_**

1	85 ± 7.07	100 ± 0	100 ± 0	100 ± 0
2	45 ± 7.07	55 ± 7.07	80 ± 14.14	100 ± 0
3	5 ± 7.07	75 ± 21.21	10 ± 0	100 ± 0
4	85 ± 7.07	70 ± 0	100 ± 0	100 ± 0
5	0 ± 0	45 ± 7.07	10 ± 14.14	100 ± 0
6	15 ± 7.07	60 ± 0	90 ± 0	100 ± 0
7	65 ± 7.07	100 ± 0	100 ± 0	100 ± 0
8	50 ± 0	60 ± 14.14	90 ± 14.14	90 ± 0
9	65 ± 7.07	90 ± 0	100 ± 0	100 ± 0
10	70 ± 0	85 ± 7.07	90 ± 0	100 ± 0
11	15 ± 7.07	90±	75 ± 7.07	100 ± 0

## Discussion

The emergence of new opportunistic fungal pathogens that are resistant to antifungal drugs is a major concern, and the WHO established a Global Antimicrobial Resistance Surveillance System (GLASS) to drive regional, national and global actions against antimicrobial resistance in the future (World Health Organization, 2019). There are many new antimicrobial resistant species emerging worldwide which include *Candida*, and among them is *C. haemulonii*, which possesses a reduced susceptibility profile to the available antifungal drugs (Ramos et al., 2015; Sanguinetti et al., 2015). New antifungal compounds are urgently required in order to circumvent this resistance profile. We have previously demonstrated the antifungal activity of copper(II), manganese(II), and silver(I) complexes containing phen-type ligands against the *C. haemulonii* complex (Gandra et al., 2017). Based on our previous results we selected one copper (1), seven manganese (2–8), and three silver chelates (9–11) for further analysis using the insect larvae, *G. mellonella*, a model widely employed as a first choice for preliminary *in vivo* antimicrobial drug testing. Initial analysis revealed that the chelates have a fungicidal activity at higher concentrations and fungistatic activity in lower doses, a profile previously described for antibiotic drugs such as fluconazole, voriconazol and caspofungin (Meletiadis et al., 2007; Venisse et al., 2008; Domán et al., 2015; Lee and Lee, 2018). A recent meta-analysis suggests that fungicidal drugs might have an advantage regarding early therapeutic success (Kumar et al., 2018), making the fungicidal properties of the chelates a possible advantageous property.

The current virulence studies using *G. mellonella* revealed that fungal inoculum size was directly associated with the infection outcome and that *C. haemulonii* was less virulent than *C. albicans*, with 10-times more *C. haemulonii* cells required to obtain similar mortality rates. This finding corroborates previous data demonstrating a higher *C. albicans* virulence to *G. mellonella* in comparison with other *Candida* species (Cotter et al., 2000; Bergin et al., 2003; Scorzoni et al., 2013). Incubation temperature also directly impacted on *Candida* virulence and mortality induced by the infection. *C. albicans* was found to be more virulent at the higher temperature of 37°C (compared to 30°C), a feature previously known for this species and other pathogenic fungi such as *Aspergillus terreus*, *Cryptococcus neoformans*, and *Cryptococcus gattii* (Scorzoni et al., 2013; Maurer et al., 2015; Rossi et al., 2016). However, *C. haemulonii* had the opposite temperature profile, with a higher mortality rate in larvae incubated at 30°C. This trend may be explained by examining the *C. haemulonii* isolation history, first from the gut of the fish *Haemulon sciurus*, and subsequently from dolphin skin and from seawater (Van Uden and Kolipinski, 1962; Gargeya et al., 1991; Khan et al., 2007). Curiously, the optimum temperature of the *H. sciurus* ranges between 25.5 and 28°C, in a median of 27.3°C (Kaschner et al., 2016). Such information could explain why, although *C. haemulonii* is capable of causing infections at 37°C, it has a higher virulence at 30°C, which could, in part, be the reason for the low incidence of this species as etiologic agents in human infections.

An inoculum-dependent fungal burden, hemocyte density and melanization processes was detected, and these aspects seem to be interrelated. For instance, while larvae infection with 10^4^ and 10^5^ cells resulted in a stable fungal burden over time, in larvae infected with 10^3^ cells/larva proliferation occurred. Such a phenomenon might be associated with the fact that the 10^3^ inoculum induced no increase in hemocytes, thus allowing the fungal cells to proliferate. Contrarily, the 10^4^ and 10^5^ inoculums resulted in increased hemocyte density and a small melanization process (10^5^), which might have kept fungal proliferation under control, since such an increase in circulating hemocytes is known to protect *G. mellonella* against fungal pathogens (Mowlds et al., 2008). Severe melanization was observed with the 10^6^ and 10^7^ inoculum, which coincided with reduced hemocyte density. This could be explained by data that suggest that, although the *G. mellonella* immune response is primarily through phagocytosis, when faced with a large number of invading microorganisms the nodulation/melanization process is mainly used by the larvae (Dunphy et al., 1986; Wu et al., 2016). Such an inoculum-dependent strategy adopted by the larvae observed in our assays was previously described in studies estimating hemocyte density in larvae inoculated with *Aspergillus fumigatus*, *C. albicans*, and *Saccharomyces cerevisiae* and in melanization studies using *C. albicans* and *C. krusei* (Bergin et al., 2006; Mowlds et al., 2010; Fallon et al., 2011a; Scorzoni et al., 2013).

All of the metal-chelates were well tolerated by *G. mellonella* in concentrations of up to 500 mg/L (10 μg/larva), but at 750 mg/L (15 μg/larva) some mortality occurred, which increased upon raising the concentration of administered chelate. Regarding the type of metal ion contained in the chelate, the solitary copper(II) complex (chelate 1) had a high toxicity profile, while the manganese(II) chelate (8) induced the lowest mortality rate. In this context, our previous study using A549 cells revealed that copper(II) chelates had very low selectivity indexes, while manganese(II) chelates demonstrated very high selectivity indexes (Gandra et al., 2017). This demonstrates that chelates containing copper(II) ions are more toxic than their manganese(II) and silver(I) counterparts toward both mammalian cells and *G. mellonella*. It is important to mention that the MIC values previously established with the chelates for all species of the *C. haemulonii* complex ranged between 0.26 and 5.96 mg/L (Gandra et al., 2017), which was much lower than the highest non-toxic concentration of all chelates (500 mg/L) observed in the present study.

The chelates, particularly the manganese(II) species (8), also demonstrated immunomodulation properties, affecting the hemocyte density and the gene expression of antimicrobial peptides (AMPs), especially *transferrin* and *IMPI*, which are key components of the larvae immune system and responsible for eliminating pathogens that have evaded the cellular response (Bergin et al., 2006). Such characteristics could be useful for a possible novel antifungal chelate, since previous studies have demonstrated that antifungal drugs, besides inhibiting proliferation and killing fungal cells, possess immunomodulatory properties that confer an additional combat mechanism against such antimicrobial resistant infections (Mesa-Arango et al., 2012). For instance, amphotericin B is capable of inducing different cell lines to produce nitric oxide (NO), prostaglandins and reactive oxygen intermediates, besides several cytokines and chemokines. Furthermore, amphotericin B, fluconazole, voriconazole, micafungin, and caspofungin are all capable of increasing the antifungal activity of different immune cells such as macrophages, polymorphonuclear leukocytes (PMN) and monocytes (Tohyama et al., 1996; Cenci et al., 1997; Chiller et al., 2001; Roilides et al., 2002; Choi et al., 2004; Ben-Ami et al., 2008; Mesa-Arango et al., 2012; Scorzoni et al., 2013; Fuchs et al., 2016). A similar increase in hemocyte density and AMPs expression in *G. mellonella* is induced by the antifungal drug caspofungin, which confers higher resistance to subsequent *C. albicans* infections (Kelly and Kavanagh, 2011). Since such immunomodulatory effects are another possible mechanism that aids the host in fighting an infection and are induced by different antifungal drugs, these immunomodulatory properties demonstrated by the chelates are an interesting trait.

Fungal burden was reduced by chelates 3, 4, 5, 7, and 8 with at least one of the concentrations used. Interestingly, all of these chelates, except the manganese(II) species (4), also induced an increase in hemocyte density in the larvae. In a previous study with larvae infected with *C. albicans* and treated with fluconazole and fluconazole associated with tetracycline-derived drugs, a similar reduction in fungal burden was reported (Gu et al., 2017). However, it is noteworthy that the drug concentrations used to achieve such an effect were much higher than the chelate concentrations used in the present study. The same study reported a gradual increase in the number of CFUs obtained from infected animals over time, indicating a limited drug activity, which a similar feature observed in the current study. Surprisingly, chelate 8 retained most of its anti-proliferation capacity, with similar numbers of CFUs obtained after 24 and 48 h of treatment. This result is important as it demonstrates that the chelates are capable of a direct *in vivo* effect and are not acting only through a secondary effect, as the immune response induced in the previous assays.

The increase in fungal burden observed with some chelates might be due to a reduction in hemocyte density or to impaired hemocyte activity when the density was not affected. With the latter scenario, our hypothesis is that the chelates might be affecting the production of reactive oxygen species (ROS) by the NADPH oxidase complex, which is essential for the larvae immune system and hemocytes to fight invading agents (Browne et al., 2013). To exemplify, it is known that fumagilin, a toxin produced by *A. fumigatus*, reduces neutrophil capacity of phagocyting and killing microorganisms due to reduced formation of the NADPH oxidase complex (Fallon et al., 2010). It has also been described that, although no reduction in *G. mellonella* or hemocyte viability was observed, fumagilin induces a reduction in hemocytes oxygen uptake, which indicates reduced ROS production since this depends on a sufficient oxygen supply besides a functioning NADPH oxidase complex. Additionally, fumagilin also reduces the hemocyte capacity of phagocyting opsonized conidia of *A. fumigatus* and opsonized *C. albicans* cells (Fallon et al., 2010, 2011b). Neutrophils and hemocytes possesses similarities regarding oxidative burst, presence of homolog proteins p40^phox^, p47^phox^, p67^phox^ e gp91^phox^ and similar microorganism elimination through the NADPH oxidase complex activity. Such information could indicate that the reduction in the hemocyte immunologic capacity occurs by a similar mechanism observed in neutrophils, the non-functionality of the NADPH complex (Fallon et al., 2011b). Knowing that chelates containing phen and related ligands are capable of uncoupling mitochondrial respiration (McCann et al., 2004), our hypothesis is that some of the present chelates are affecting the formation or activity of the NADPH oxidase complex, and interfering with the ROS formation capacity of hemocytes, reducing its phagocytosis and killing capacity.

Finally, a dramatic reduction in larvae mortality was observed when *C. haemulonii* cells were exposed to chelates 2, 3, 5, 6, 8, and 11 prior to infection of *G. mellonella* larvae. Such an outcome is known as the post-antifungal effect (PAFE), which is described as an inhibition of fungal proliferation after a limited exposure to an antifungal agent (Minguez et al., 1994). Such an attenuated virulence of *C. albicans*, *Candida Tropicalis*, and *Candida kefyr* was previously reported for these fungal cells upon exposure to sub-inhibitory concentrations of amphotericin B, fluconazole, ketoconazole, and 5-flucitosine, which affected aspects such as growth rate, susceptibility to PMN leukocytes and adhesion capacity to buccal epithelial cells (Abu-El Teen et al., 1989; Minguez et al., 1994, 1997). The capacity of the metal-chelates to affect *C. haemulonii* virulence in a similar way to classical antifungal drugs reinforces the possible potential of using such compounds as antifungal agents.

In summary, our data indicates that manganese(II) and silver(I) chelates containing the phen ligand have potent anti-*C. haemulonii* activity and are reasonably non-toxic toward *G. mellonella*. Variations in toxicity levels and on the immune response of larvae exposed to the chelates indicates that chemical structural differences, even minimal ones, directly impacts over how the chelates affect the fungal cells and the host, *G. mellonella*. The manganese(II) chelate, 8, demonstrated very interesting characteristics, possessing very good antifungal capacity, low toxicity, a capacity to induce an immune response and to reduce the fungal burden on infected animals and also an ability to affect *C. haemulonii* virulence. More research is needed in order to better understand the mechanisms of action of the chelates and to allow for the development of new compounds that are more active and less toxic and which could become alternative therapeutic agents for treating multi-resistant *Candida* species. We believe that the present study offers important insights for the future treatment of antimicrobial resistant pathogens worldwide.

## Data Availability Statement

All datasets generated for this study are included in the article/supplementary material.

## Author Contributions

RG, LV, KK, AS, OH, and MD conceived and designed the study. RG, PM, and MF performed the experiments. All authors analyzed the data. MM, MB, AS, OH, and MD contributed reagents, materials, and/or analysis tools. RG, AS, MM, OH, and MD wrote and revised the manuscript. All authors contributed to the research and approved the final version of the manuscript. All authors agreed to be accountable for all aspects of the work.

## Conflict of Interest

The authors declare that the research was conducted in the absence of any commercial or financial relationships that could be construed as a potential conflict of interest.
